# Structure-based virtual screening of highly potent inhibitors of the nematode chitinase *Ce*Cht1

**DOI:** 10.1080/14756366.2021.1931862

**Published:** 2021-06-02

**Authors:** Wei Chen, Qi Chen, Ashutosh Kumar, Xi Jiang, Kam Y. J. Zhang, Qing Yang

**Affiliations:** a State Key Laboratory for Biology of Plant Diseases and Insect Pests, Institute of Plant Protection, Chinese Academy of Agricultural Sciences, Beijing, China; b Laboratory for Structural Bioinformatics, Center for Biosystems Dynamics Research, RIKEN, Yokohama, Japan; c Guangdong Laboratory for Lingnan Modern Agriculture (Shenzhen Branch), Agricultural Genomics Institute at Shenzhen, Chinese Academy of Agricultural Sciences, Shenzhen, China; d School of Bioengineering, Dalian University of Technology, Dalian, China

**Keywords:** Nematode chitinase, inhibitor, inhibitory mechanism, structural optimisation

## Abstract

Nematode chitinases play vital roles in various physiological processes, including egg hatching, larva moulting, and reproduction. Small-molecule inhibitors of nematode chitinases have potential applications for controlling nematode pests. On the basis of the crystal structure of *Ce*Cht1, a representative chitinase indispensable to the eggshell chitin degradation of the model nematode *Caenorhabditis elegans*, we have discovered a series of novel inhibitors bearing a (*R*)-3,4-diphenyl-4,5-dihydropyrrolo[3,4-*c*]pyrazol-6(2*H*)-one scaffold by hierarchical virtual screening. The crystal structures of *Ce*Cht1 complexed with two of these inhibitors clearly elucidated their interactions with the enzyme active site. Based on the inhibitory mechanism, several analogues with improved inhibitory activities were identified, among which the compound **PP28** exhibited the most potent activity with a *K*_i_ value of 0.18 μM. This work provides the structural basis for the development of novel nematode chitinase inhibitors.

## 1. Introduction

GH18 chitinases hydrolyse β-1,4-glycosidic bonds in chitin and chitooligosaccharides. As chitin is present in the eggshell, cuticle, pharynx and microfilarial sheath of nematodes^1–5^, nematode chitinases have been shown to play an important role in various physiological processes, including egg hatching, larva moulting, and reproduction^6–8^. Downregulating the expression level of nematode chitinases led to hatching delay and moulting defects in many nematode species, such as the free-living model nematode *Caenorhabditis elegans*, the plant parasitic nematode *Bursaphelenchus xylophilus*, and the animal parasitic nematodes *Acanthocheilonema viteae* and *Onchocerca volvulus*^9–11^.

The importance of nematode chitinases indicates that they may be promising nematicide targets for the development of small-molecule inhibitors for nematode pest control^12^. Many GH18 chitinase inhibitors with diverse scaffolds have been reported so far, and some showed potential applications as antifungal agents, pesticides, and drugs^6^^,^^13–15^. However, the inhibition of nematode chitinases is rarely studied, and only few inhibitors have been reported to be effective on nematode chitinases, including allosamidin, closantel, β-carboline, and 4-hydroxy-1,2,3-triazoles. Allosamidin, a natural product derived from the mycelium of *Streptomyces* sp., is a broad-spectrum GH18 chitinase inhibitor^16^. As a substrate analogue, allosamidin showed inhibitory activity against nematode chitinases from *Heligmosomoides polygyrus*, *Brugia malayi*, *Loa*, and *Wuchereria bancrofti*^10^^,^^17^. Allosamidin could also retard egg hatching and inhibited exsheathment. However, the polysaccharide scaffold of allosamidin makes it difficult to synthesise and has poor druggability. Closantel, a known anthelmintic drug, was previously discovered as a potent inhibitor against *Ov*Cht1 from *O. volvulus* and *Bm*Cht1 from *B. malayi*^18^. Closantel and its derivates were capable of affecting *O. volvulus* L3 molting^19–21^. In continued studies to discover *Ov*Cht1 inhibitors, 4-hydroxy-1,2,3-triazoles were identified through bioisosteric modulation and scaffold hopping approaches^22^, and β-carbolines were obtained by screening a commercial library of natural products^23^. β-carbolines were capable of penetrating the worm cuticle and preventing filaria moulting. However, the binding modes of these compounds have not been elucidated, which imposes restrictions on their further optimisation and application.

The scarce nematode chitinase inhibitors may be, to a great extent, attributed to the lagged research on the structure of nematode chitinases. The availability of structure information could facilitate both structure-based virtual screening for inhibitor development and elucidation of inhibitory mechanism for inhibitor optimization^24–27^. Recently, we resolved the crystal structure of *Ce*Cht1 (PDB ID: 6LDU), a chitinase from the model nematode *C. elegans*^28^. In this study, exploiting the structure of *Ce*Cht1, we identified a series of inhibitors bearing a (*R*)-3,4-diphenyl-4,5-dihydropyrrolo[3,4-*c*]pyrazol-6(2*H*)-one (**PP**) scaffold. In addition, we demonstrated the binding mechanism by X-ray crystallographic analysis, which facilitated the further optimisation of these compounds and led to the identification of several compounds with improved inhibitory activity. This work provides a solid basis for the development of nematode chitinase inhibitors.

## 2. Materials and methods

### 2.1. Protein expression and purification

The DNA encoding the target protein with a C-terminal 6 × His affinity tag was cloned into pPIC9 vector and transformed into *Pichia pastoris* GS115 (Invitrogen, Carlsbad, CA). After 120 h of fermentation, the culture supernatant was collected and subjected to ammonium sulphate precipitation. The precipitate was dissolved and purified with a HisTrap FF affinity column (GE Healthcare, Uppsala, Sweden). Then the protein was deglycosylated by PGNase F and the deglycosylase was removed through HisTrap FF affinity column. The protein was further purified by anion-exchange chromatography.

### 2.2. Virtual screening

A hierarchical virtual screening strategy was used as described previously^13^^,^^27^^,^^29^. First, structural analogues to active hits were identified from a subset of commercially available compounds from ZINC database^30^ employing substructure search and shape similarity calculations. Substructure search was performed using OEChem toolkit (OpenEye Scientific Software, Santa Fe, NM). Shape similarity calculations were performed using ROCS^31^. The conformational database used for shape similarity calculations was prepared using OMEGA^32^. Compounds in the screening library were scored using “TanimotoCombo” score. Structural analogues were then prioritised for the evaluation of *Ce*Cht1 inhibitory activity using molecular docking. The crystal structure of *Ce*Cht1-CAD was used for molecular docking calculations. The protein structure for molecular docking was prepared using Maestro, where all water molecules were removed, hydrogens were added and protonation states of all charged residues were assigned at neutral pH. Ligands for molecular docking were prepared using LigPrep. Tautomeric and ionisation states of all ligands were determined using Epik program^33^ at neutral pH. Molecular docking was performed using Glide program in extra precision mode^34–36^. Grids for molecular docking calculation were prepared by including the catalytic residues and residues in both “+” and “−” GlcNAc binding subsites. Ligands were scored using Glidescore with Epik penalties and a single pose per compound was saved.

### 2.3. Inhibitory activity assays

Compounds selected by virtual screening were purchased from Topscience (Shanghai, China; http://www.tsbiochem.com) for inhibitory activity assays. The inhibitory activity were assayed in end-point experiments using 4-methylumbelliferyl β-d-*N,N'*-diacetylchitobioside hydrate (4MU-(GlcNAc)_2_, Sigma, St. Louis, MO) as a substrate. The reaction mixture containing 20 mM sodium phosphate buffer (pH 6.0), 1% (v/v) DMSO, 10 nM *Ce*Cht1-CAD, 4 μM 4MU-(GlcNAc)_2_ and inhibitor was incubated in a final volume of 100 μL at 25 °C for 20 min. The reaction was stopped by adding 100 μL 0.5 M sodium carbonate, and fluorescence of the released 4-MU was quantified (excitation 366 nm, emission 440 nm). Experiments were performed in triplicate unless otherwise specified. The inhibition constant (*K*_i_) was calculated using Dixon plots by changing the compound concentration at several fixed concentrations of 4MU-(GlcNAc)_2_ (2 μM, 4 μM, and 8 μM).

### 2.4. Crystallisation, data collection, and structure determination

The purified protein was desalted in a buffer containing 20 mM Tris-HCl (pH 7.5), 20 mM NaCl and spin-concentrated to 15.0 mg/mL. For crystallisation of *Ce*Cht1 with bound inhibitors, the protein was incubated with inhibitor at a final concentration of 0.1 mM overnight. Then co-crystallisation experiments were performed by vapour diffusion in hanging drops at 4 °C. The volume ratio of protein to reservoir was 1:1 and the reservoir solution contained 0.1 M Bis-Tris, pH 6.0, and 25% PEG3350. Crystals were cryoprotected by gently increasing the cryoprotectant concentration in the drops (up to 22% glycerol) and directly flash frozen by immersion in liquid nitrogen before data collection.

The diffraction data were collected on the BL18U1 and BL19U1 at the Shanghai Synchrotron Radiation Facility in China^37^, and the diffraction data were processed using the *HKL-3000* package^38^. Structures were determined by molecular replacement with *Phaser* using native *Ce*Cht1 (PDB ID: 6LDU) as the search model^39^. Iterative molecular models were manually built and extended using *Coot*^40^, and the X-ray structure was refined by PHENIX suite of programs^41^. Structural figures were prepared by PyMOL (DeLano Scientific, San Carlos, CA). The data collection and structure refinement statistics are summarised in Table 2.

## 3. Results and discussion

### 3.1. Identification of CeCht1 inhibitors with novel scaffold

**Figure 1.**
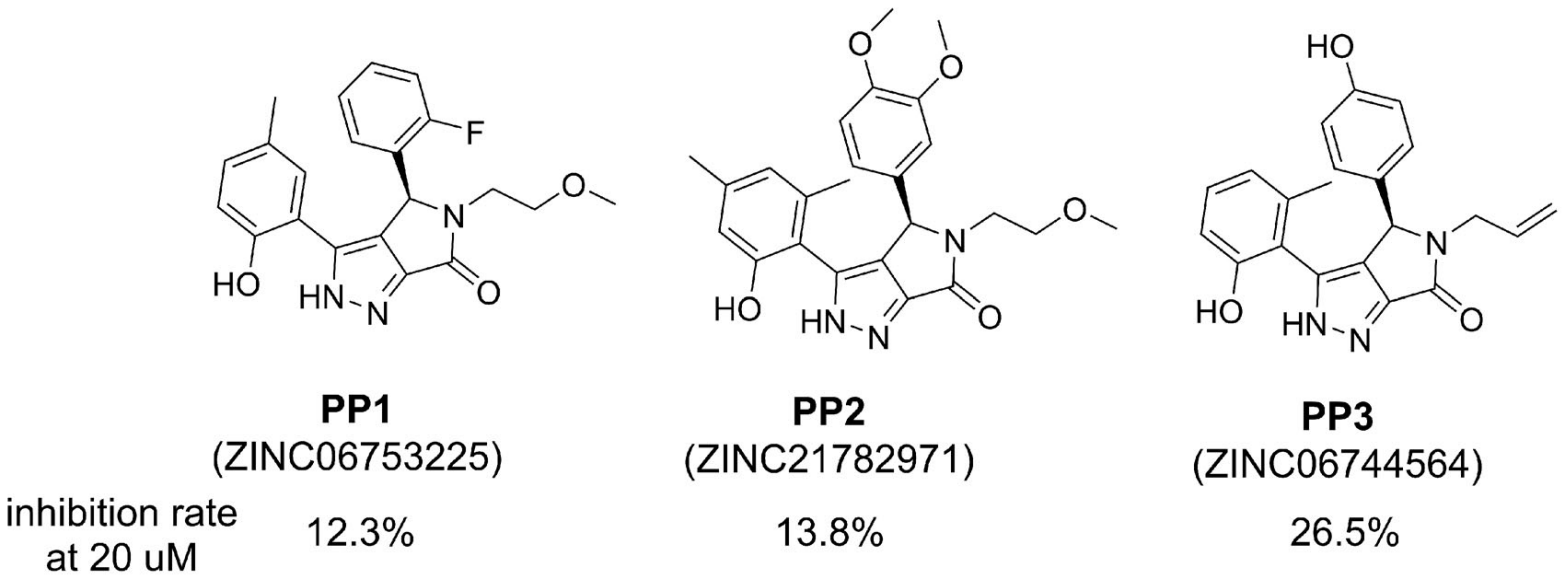
Structure of lead compounds bearing a (*R*)-3,4-diphenyl-4,5-dihydropyrrolo[3,4-*c*]pyrazol-6(2*H*)-one scaffold.

**Figure 2.**
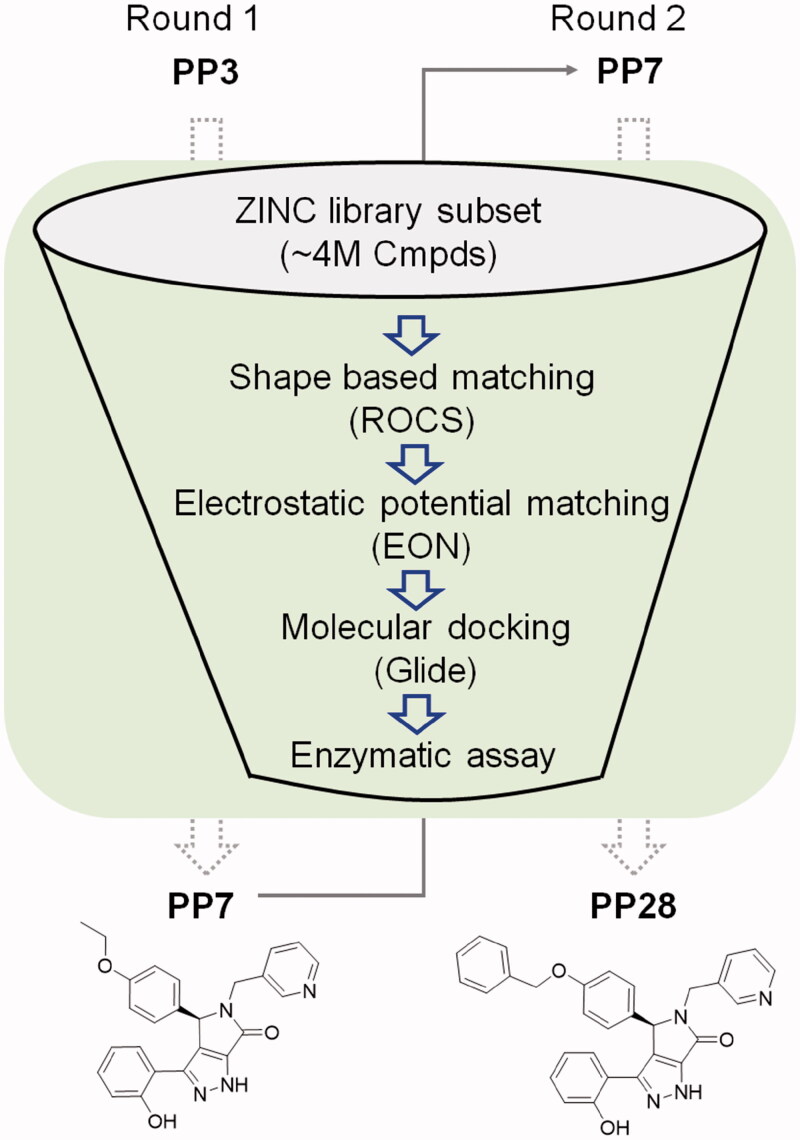
Hierarchical virtual screening strategy. A combination of shape similarity calculations, electrostatic potential similarity calculations, and molecular docking was used to identify compounds for the enzymatic assay.

Screening of an in-house collection of compounds accumulated in various chitinase inhibitor discovery projects in our laboratory resulted in the identification of several compounds that showed moderate *Ce*Cht1 inhibitory activity. Among these compounds, there were three compounds bearing a similar (*R*)-3,4-diphenyl-4,5-dihydropyrrolo[3,4-*c*]pyrazol-6(2*H*)-one scaffold (Figure 1), and the best one (**PP3)** inhibited *Ce*Cht1 with a *K*_i_ value of 38.3 μM (Table 1). As compounds with this scaffold have not been previously described to possess activity against any chitinase, we decided to proceed with compound **PP3**. To identify compounds with improved *Ce*Cht1 inhibition, a hierarchical virtual screening was performed (Figure 2). Initially, structural analogues were identified employing substructure search and shape similarity calculations using compound **PP3** as the starting structure. Finally, molecular docking was used to prioritise compounds for the evaluation of *Ce*Cht1 inhibitory activity. A set of compounds (**PP4**–**PP26**) were identified, and most of these compounds showed improved inhibitory activity over the starting compound and the reported inhibitor closantel (Table 1).

**Table 1.** Inhibitory activity of **PP3**–**PP26** against *Ce*Cht1.


**Compound**	R_1_	R_2_	R_3_	R_4_	R_5_	*K*_i_ (μM)
**PP3** (ZINC06744564)	−CH = CH_2_	H	−OH	H	H	38.32 ± 4.21
**PP4** (ZINC09408925)		H	−OCH_2_C_6_H_5_	H	H	46.39 ± 3.97
**PP5** (ZINC09408989)		H	−O(CH_2_)_2_CH_3_	H	H	51.28 ± 2.02
**PP6** (ZINC08606651)		H	−OCH_2_CH_3_	H	H	6.03 ± 0.16
**PP7** (ZINC08845352)		H	−OCH_2_CH_3_	H	H	0.76 ± 0.06
**PP8** (ZINC09124438)		H	−OCH_2_CH_3_	−CH_3_	−CH_3_	15.42 ± 2.04
**PP9** (ZINC06744628)		H	−OCH_3_	H	H	2.27 ± 0.04
**PP10** (ZINC09243696)		H	−OCH_3_	−CH_3_	−CH_3_	13.68 ± 0.64
**PP11** (ZINC08845718)		H	−OCH_3_	−CH_3_	H	ND
**PP12** (ZINC08845425)		H	−SCH_3_	H	H	1.11 ± 0.05
**PP13** (ZINC06744597)		H	−OH	H	H	22.08 ± 1.30
**PP14** (ZINC06040091)		H	−CH_3_	H	H	5.69 ± 0.72
**PP15** (ZINC06744580)		H	−F	H	H	20.51 ± 0.17
**PP16** (ZINC06744570)		H	−Cl	H	H	2.35 ± 0.09
**PP17** (ZINC08845344)		H	−Br	H	H	2.24 ± 0.13
**PP18** (ZINC16806320)		H	−Cl	−CH_3_	−CH_3_	27.62 ± 2.21
**PP19** (ZINC08845720)		−OCH_3_	H	−CH_3_	H	ND
**PP20** (ZINC08845776)		−OCH_3_	−OCH_3_	−CH_3_	H	ND
**PP21** (ZINC08845462)		−OCH_3_	−O(CH_2_)_2_CH_3_	H	H	4.51 ± 0.98
**PP22** (ZINC08845314)		−OCH_3_	−OCH_2_C_2_H_3_	H	H	17.72 ± 2.16
**PP23** (ZINC11914967)		−OCH_3_	−OCH_2_CH_3_	−CH_3_	H	ND
**PP24** (ZINC09243425)		−OCH_3_	−OCH_2_CH_3_	−CH_3_	−CH_3_	19.64 ± 0.18
**PP25** (ZINC06744675)		−OH	H	H	H	23.52 ± 1.51
**PP26** (ZINC06753267)		−OH	H	−CH_3_	H	ND
**Closantel**^a^	−	−	−	−	−	9.02 ± 1.01

ND: not determined (less than 50% inhibition at 50 μM).

^a^The reported nematode chitinase inhibitor closantel is used as the positive control.

### 3.2. Structure–activity relationship analysis

As shown in Table 1, all the compounds with better activity than **PP3** had a pyridine group at R_1_ position, indicating that the increase of hydrophobicity in this position may be of benefit to the inhibitory activity. It is worth noting that the position of the nitrogen atom in pyridine group had a marked impact on the inhibitory activity because the inhibitory activity of **PP7** increased nearly 10-fold over **PP6**. The *para*-substitution was obviously superior to the *meta*-substitution, suggesting that the nitrogen atom may form important interactions with *Ce*Cht1. The substituent at R_2_ position seemed to have little effect on the inhibitory activity as compounds **PP8** and **PP24** exhibited similar *K*_i_ values. Compounds **PP7**, **PP9**, and **PP12**–**PP17** only differed in the R_3_ position, but their inhibitory activities showed significant difference. A bulky group at R_3_ position may facilitate the increase of inhibitory activity. Comparison of **PP9**–**PP11** or **PP22**–**PP24** revealed that the methyl substituent at R_4_ and R_5_ positions is not conducive to inhibit *Ce*Cht1, especially for a mono-substitution at R_4_ position. The differences in bioassay results between **PP7** and **PP8**, or **PP25** and **PP26**, also supported this inference.

### 3.3. Inhibitory mechanism

To gain molecular insights into the inhibitory mechanism of the **PP** series of compounds, we solved the structures of *Ce*Cht1 in complex with two inhibitors, **PP7** and **PP21** (Table 2), which potently inhibited *Ce*Cht1 with *K*_i_ values of 0.76 μM and 4.51 μM, respectively.

**Table 2.** X-ray data collection and structure-refinement statistics

	*Ce*Cht1–**PP7**	*Ce*Cht1–**PP21**
Protein Data Bank entry	6LE8	6LE7
Space group	P22_1_2_1_	P12_1_1
*Unit-cell parameters*		
*a*, *b*, *c* (Å)	54.25, 54.73, 139.85	47.63, 67.12, 57.06
*α*, *β*, *γ* (°)	90.00, 90.00, 90.00	90.00, 103.66, 90.00
Wavelength (Å)	0.97854	0.97852
Temperature (K)	100	100
Resolution (Å)	50.00–1.40 (1.45–1.40)	50.00–1.86 (1.93–1.86)
Unique reflections	81,919 (7874)	28,692 (2710)
Observed reflections	1,059,913	181,761
*R*_merge_	0.088 (0.826)	0.134 (0.597)
Average multiplicity	12.9 (11.9)	6.3 (6.7)
*I*/σ(*I*)	11.819 (1.912)	13.056 (2.917)
Completeness (%)	98.8 (96.9)	97.6 (97.1)
*R*/*R*_free_	0.1549/0.1668	0.2163/0.2571
Protein atoms	2986	2978
Water molecules	604	277
Other atoms	52	35
*R.m.s. deviation from ideal*		
Bond lengths (Å)	0.006	0.012
Bond angles (°)	0.87	1.18
Wilson B factor (Å^2^)	13.25	26.28
Average B factor (Å^2^)	16.31	29.82
Protein atoms	14.00	29.25
Water molecules	27.85	34.83
Ligand molecules	15.15	35.65
*Ramachandran plot* (%)		
Favoured	98.7	97.6
Allowed	1.3	2.4
Outliers	0	0

**Figure 3.**
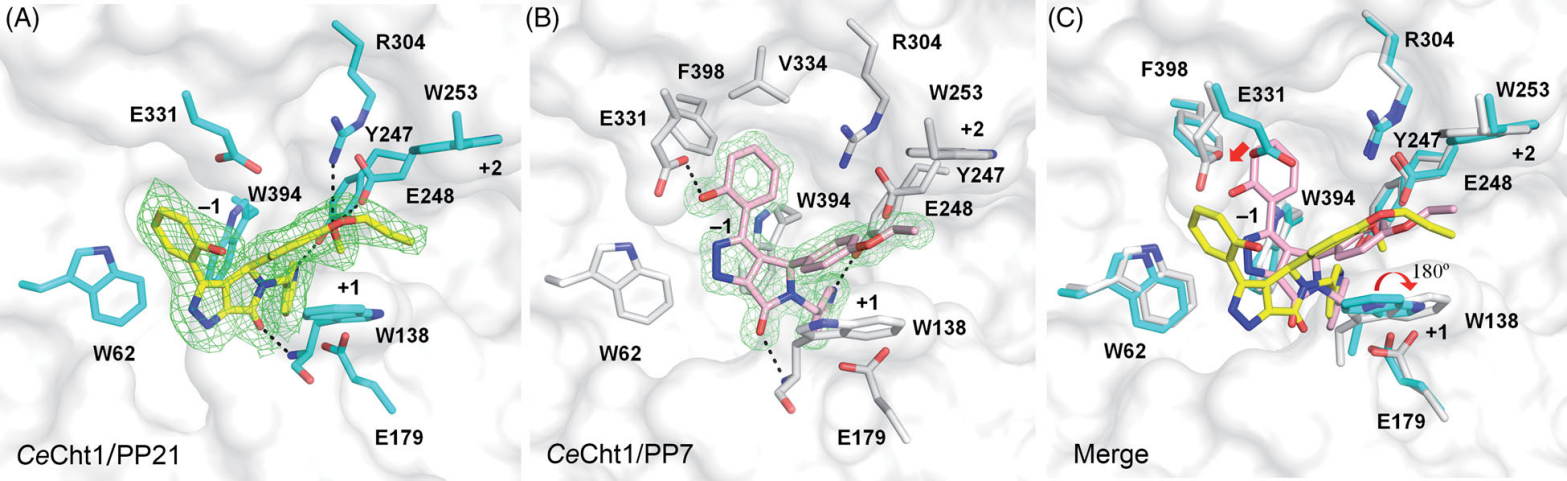
Interactions between inhibitors and *Ce*Cht1. (A, B) The binding conformation of **PP21** and **PP7** are shown in sticks with yellow and pink carbon atoms, respectively. The 2Fo-Fc electron density map around the ligand is contoured at the 1.0*σ* level and shown as green mesh. The residues of *Ce*Cht1 participating in the interactions with each inhibitor are labelled with residue numbers and shown in cyan (A) and grey (B) sticks, respectively. Hydrogen bonds are displayed as dashed lines. The numbers indicate the subsite of substrate-binding cleft. (C) Merged view of the active site region of *Ce*Cht1.

The crystal structure of *Ce*Cht1 in complex with **PP21** was determined at a resolution of 1.86 Å. The electron density map showed that **PP21** was well-anchored in the substrate-binding cleft of *Ce*Cht1 from subsites −1 to +2, and stabilised by hydrophobic interactions and hydrogen bonds (Figure 3(A)). The nomenclature for substrate-binding subsites was named according to Davies et al., where subsite –n represents the non-reducing end and subsite + n represents the reducing end^42^. The structure provided an explanation for the above structure–activity analysis. The dihydropyrrolopyrazol-6-one skeleton bound in a hydrophobic pocket lined with several aromatic residues and formed a 2.5-Å hydrogen bond with the backbone of Trp138 at the +1 subsite. The pyridine moiety penetrated into the active site pocket and stacked well with Trp394 at the −1 subsite. Besides, the nitrogen atom formed a hydrogen bond with Tyr247. These interactions elucidated the reasons why pyridine group at R_1_ position could significantly increase inhibitory activity. The phenol moiety interacted with Trp62 via T-shaped π–π contacts, and methyl substituents at R_4_ and R_5_ positions of the benzene ring would cause steric hindrance, resulting in a decrease of inhibitory activity. The 3-methoxy-4-propoxyphenyl moiety of **PP21** interacted with Trp138 with hydrophobic contacts. The methoxy group formed hydrogen bonds with Asp248 and Arg304, while the propoxy group extended to Trp253 at the +2 subsite.

**PP7** is the most potent among these compounds, with a *K*_i_ value of 0.76 μM. The structure of the complex was also obtained and refined to 1.40 Å. The electron density map of the ligand was unambiguous in the substrate-binding cleft, which could easily be used to reconstruct the conformation of **PP7** and clearly showed details of the interactions (Figure 3(B)). The dihydropyrrolopyrazol-6-one skeleton of **PP7** was anchored in the hydrophobic pocket and formed a hydrogen bond with Trp138 while the pyridine moiety interacted with Trp394 and Tyr247, which was similar with those observed in **PP21**–*Ce*Cht1 complex. The phenol moiety bound in a small hydrophobic cave constructed by residues Val334, Tyr302, and Phe398, and it was further stabilised by forming a 2.6-Å hydrogen bond with Glu331. The ethoxyphenyl moiety hydrophobically interacted with Trp138.

The main chemical structure difference between **PP21** and **PP7** was the substitution at R_4_ position. Compared with **PP7**, **PP21** had a methoxy at R_4_ position, which led to a 6.7-fold decrease of the *K*_i_ value. Structural comparison showed differences in the binding modes of these two inhibitors (Figure 3(C)). First, although the lack of a methoxy group at R_4_ position of **PP7** abolished the formation of hydrogen bonds with Asp248 and Arg304, it pulled the inhibitor closer to the active site pocket. As a result, the side chain of Trp138 rotated 180° and stacked well with the ethoxyphenyl moiety of **PP7**. Second, the dihydropyrrolopyrazol-6-one skeleton of **PP7** rotated about 51° and also got closer to the protein. This rotation resulted in a bigger conformation change of the phenol moiety which formed more stable hydrophobic contacts and induced a conformation shift of Glu331 to form a hydrogen bond. Finally, the dihydropyrrolopyrazol-6-one skeleton and phenol moiety was coplanar, which could enhance the hydrophobic interactions between **PP7** and *Ce*Cht1. Therefore, these structural differences made **PP7** a more potent inhibitor than **PP21**.

### 3.4. Structure-guided discovery of more potent inhibitors

**Figure 4.**
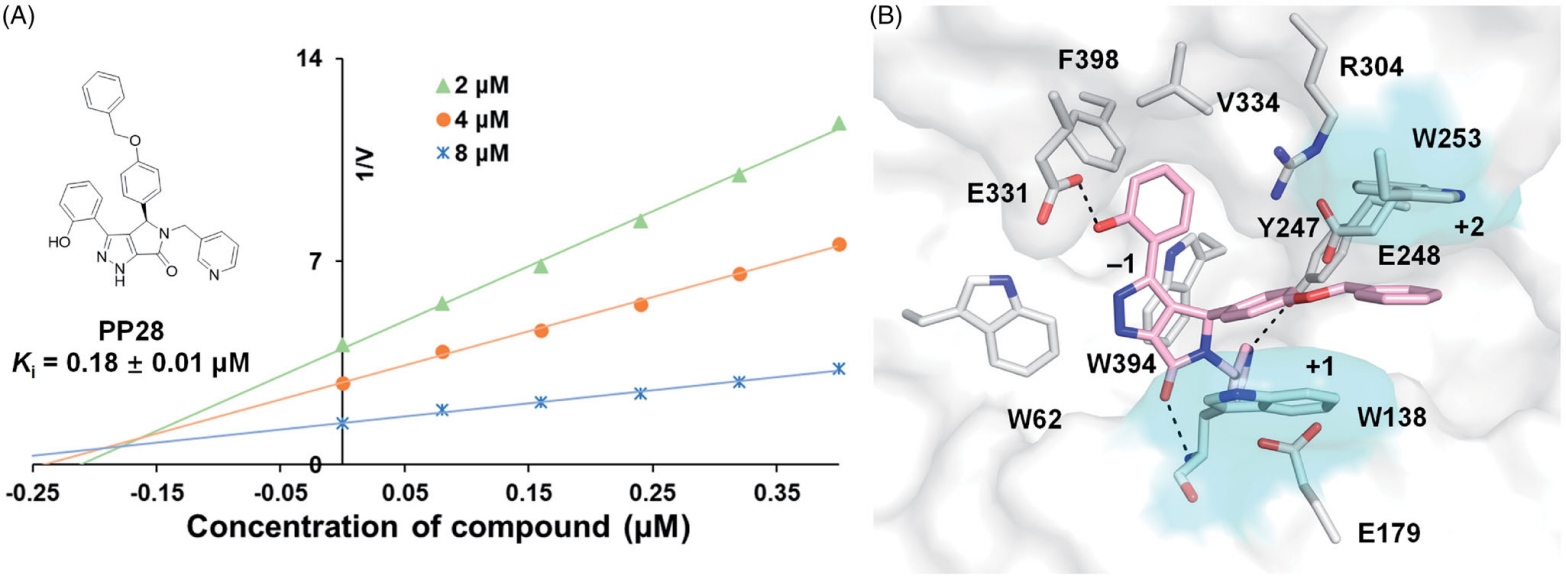
Inhibitory activity and mechanism of compound **PP28** against *Ce*Cht1. (A) Inhibition kinetics of **PP28** against *Ce*Cht1 were determined by Dixon plot analysis. (B) Molecular docking of *Ce*Cht1 in complex with **PP28**. The ligands are shown as sticks with pink carbon atoms. The two important tryptophans that interact with the compound are shown as sticks with cyan carbon atoms.

Structure–activity relationship analyses indicated that a bulky group at R_3_ position is beneficial to the increase of inhibitory activity. The two crystal structures of inhibitor complexes showed that both the R_3_ group (the propoxy group of **PP21** and the ethoxy group of **PP7**), extended to the edge of Trp253 at the +2 subsite. However, the interaction between these inhibitors and Trp253 was weak and there was still a plenty of space to accommodate a bigger group. Therefore, we hypothesised that **PP7** derivatives with bulkier substituent groups at R_3_ position would have better inhibitory activity. To confirm this hypothesis, we performed another round of virtual screening and obtained several derivatives (**PP27**–**PP32**). The inhibitory activity analysis showed that four compounds were better than **PP7** (Table 3). Among these, compound **PP28** exhibited the most potent activity as a competitive inhibitor, with a *K*_i_ value of 0.18 μM (Figure 4(A)), which was a 4-fold increase than that of **PP7**. The docking calculation indicated that the benzyloxy phenyl group of **PP28** extended to the cavity between Trp138 and Trp253 (Figure 4(B)). The benzyloxy phenyl group hydrophobically interacted with Trp253 and Trp138, forming a sandwich structure, which further improved the affinity of **PP28**. Besides, these two tryptophans together with the cavity they formed are conserved among different GH18 chitinases, and many GH18 chitinase inhibitors have taken advantage of this structural feature. Therefore, further optimisation at this position in the compounds might lead to better inhibitors.

**Table 3.** Inhibitory activity of **PP27**−**PP32** against *Ce*Cht1


Compound	R	*K*_i_ (μM)
**PP7** (ZINC08845352)		0.76 ± 0.06
**PP27** (ZINC27664561)		0.19 ± 0.02
**PP28** (ZINC38609907)		0.18 ± 0.01
**PP29** (ZINC08606647)		0.33 ± 0.02
**PP30** (ZINC08606645)		0.55 ± 0.04
**PP31** (ZINC08845437)		1.01 ± 0.10
**PP32** (ZINC08845431)		1.37 ± 0.07

## 4. Conclusion

In summary, we have identified a series of *Ce*Cht1 inhibitors bearing a novel scaffold. Structure–activity relationship analyses and crystallography studies clearly elucidated the inhibitory mechanism of these compounds. The crystal structures of enzyme–inhibitor complexes provided clues to develop compounds with improved inhibitory activity. This work presents an efficient strategy, which combined computational and experimental studies, to discover potent inhibitors. In addition, this work may promote further development of nematicides to deal with the increasing damages caused by nematode pests.
